# Android Ransomware Detection Using Supervised Machine Learning Techniques Based on Traffic Analysis

**DOI:** 10.3390/s24010189

**Published:** 2023-12-28

**Authors:** Amnah Albin Ahmed, Afrah Shaahid, Fatima Alnasser, Shahad Alfaddagh, Shadha Binagag, Deemah Alqahtani

**Affiliations:** 1 Department of Computer Science, College of Computer Science and Information Technology, Imam Abdulrahman Bin Faisal University, P.O. Box 1982, Dammam 31441, Saudi Arabia; aabinahmad@iau.edu.sa (A.A.A.); 2190009057@iau.edu.sa (A.S.); 2190005999@iau.edu.sa (S.B.); 2 SAUDI ARAMCO Cybersecurity Chair, Department of Computer Science, College of Computer Science and Information Technology, Imam Abdulrahman Bin Faisal University, P.O. Box 1982, Dammam 31441, Saudi Arabia

**Keywords:** android security, ransomware attacks, cyber-attacks, machine learning, deep learning, ensemble learning

## Abstract

In today’s digitalized era, the usage of Android devices is being extensively witnessed in various sectors. Cybercriminals inevitably adapt to new security technologies and utilize these platforms to exploit vulnerabilities for nefarious purposes, such as stealing users’ sensitive and personal data. This may result in financial losses, discredit, ransomware, or the spreading of infectious malware and other catastrophic cyber-attacks. Due to the fact that ransomware encrypts user data and requests a ransom payment in exchange for the decryption key, it is one of the most devastating types of malicious software. The implications of ransomware attacks can range from a loss of essential data to a disruption of business operations and significant monetary damage. Artificial intelligence (AI)-based techniques, namely machine learning (ML), have proven to be notable in the detection of Android ransomware attacks. However, ensemble models and deep learning (DL) models have not been sufficiently explored. Therefore, in this study, we utilized ML- and DL-based techniques to build efficient, precise, and robust models for binary classification. A publicly available dataset from Kaggle consisting of 392,035 records with benign traffic and 10 different types of Android ransomware attacks was used to train and test the models. Two experiments were carried out. In experiment 1, all the features of the dataset were used. In experiment 2, only the best 19 features were used. The deployed models included a decision tree (DT), support vector machine (SVM), k-nearest neighbor (KNN), ensemble of (DT, SVM, and KNN), feedforward neural network (FNN), and tabular attention network (TabNet). Overall, the experiments yielded excellent results. DT outperformed the others, with an accuracy of 97.24%, precision of 98.50%, and F1-score of 98.45%. Whereas, in terms of the highest recall, SVM achieved 100%. The acquired results were thoroughly discussed, in addition to addressing limitations and exploring potential directions for future work.

## 1. Introduction

The rapid growth in the use of mobile devices has made Android one of the leading operating systems for smartphones and tablets. As of July 2023, Android has the highest market share of 70.8% [1]. Android is an open-source, Linux-based mobile operating system developed by Google [2]. Android 13 is the most recent version, released in 2022. It supports many technologies such as Wi-Fi, short message service (SMS), Bluetooth, accelerometers, camera, global positioning systems (GPS), voice over LTE (VoLTE), etc. Because of its open nature, Android has become immensely popular among developers and consumers. Additionally, software developers can quickly alter and upgrade it to conform to the most recent standards for mobile technology. Unfortunately, this popularity has also attracted cybercriminals who exploit users’ private data and personal information without their consent [3]. One of the most prevalent and disruptive attacks targeting Android devices is ransomware. Ransomware constitutes a type of malware that encrypts files on a device and demands payment for their decryption. Typically, cybercriminals demand payment in cryptocurrencies such as Bitcoin to evade detection. A typical scenario in ransomware attacks often begins when the user downloads a fraudulent application from either the Google Play Store or an alternative third-party marketplace [4]. With the increasing presence of Android devices and the open nature of the platform, which allows easy app downloads from unofficial sources, the severity of ransomware attacks has reached alarming levels. In such a situation, data recovery can be challenging, and the risk of further attacks and identity theft is increased, leading to a diminished trust in security solutions by users [5,6]. Ransomware attacks commonly focus on specific industries. In 2022, manufacturing companies worldwide experienced 437 such attacks, while the food and beverage sector followed closely with more than 50 ransomware incidents. When it comes to the distribution of ransomware attacks on critical infrastructure, North America took the lead among global regions, with Europe following in second place [7]. Additionally, the global number of ransomware attacks per year from 2017 to 2022 is as follows: in 2022, organizations detected a staggering 493.33 million ransomware attacks worldwide [7].

Moving forward to how ransomware is carried out in Android devices; firstly, it is important to know that ransomware continually grows with advanced encryption capabilities by displacing established standards, such as phishing, banking trojans, distributed denial-of-service (DDoS), and crypto-jacking. However, criminals use these models as an initial stepping-stone, then escalate the attack and eventually carry out the targeted attacks, hence forcing payment from victims. By opening an email attachment or clicking an ad, accessing a link, or even navigating to a website that has malware embedded in it, one can unknowingly download ransomware onto an electronic device. Once the code has been loaded on a device, it locks access to the device and any stored files and data. Versions that are more destructive can encrypt data on networked devices as well as local drives and attached drives. The users discover it when their data become inaccessible, or when messages pop up informing them of the attack and demanding ransom payments. Criminals threaten to publicly expose confidential data if their victims do not pay within the specified time frame or opt to recover encrypted data through backups. Some attackers even sell confidential data at auction on the dark web. It is essential to know that ransomware attacks have numerous different appearances, and they show up in all shapes and sizes. There are two main categories, namely lock-screen and crypto [8]. In the lock screen, the ransomware blocks access to the system, asserting that the system is encrypted. Lock-screen ransomware does not usually target critical files; it generally only wants to lock the user out. On the other hand, crypto ransomware encrypts data on a system, such as documents, pictures, and videos, making the content useless without the decryption key and without interfering with basic device functions. Users can see their files but cannot access them unless they pay the ransom demand, or all their files would be deleted. Some examples of Android ransomware include Android/Simplocker, Android/Lockerpin, WannaLocker, etc. [8]. Consequently, there is a dire need to develop effective methods for detecting Android ransomware to counter this escalating danger.

Android devices come equipped with a variety of embedded sensors that measure motion, orientation, and other environmental factors. The embedded sensors are managed by the Android sensor framework. Sensors are either hardware-based or software-based. Hardware-based sensors are physical components that are integrated into a tablet or phone. They acquire their data by directly sensing certain parameters. Software-based sensors are not physical devices. Software-based sensors, also referred to as virtual or synthetic sensors, obtain their data from one or more hardware-based sensors. Three major categories of sensors (motion, environmental, position) are supported by the Android operating system. Motion sensors measure rotational force and acceleration force in three dimensions. Rotational vector sensors, gyroscopes, accelerometers, and gravity sensors are included in this category. Environmental sensors monitor a range of environmental factors, such as humidity, illumination, ambient air pressure, and temperature. Thermometers, photometers, and barometers fall under this category. Position sensors measure the physical position of a device. Magnetometers and orientation sensors fall under this category. Android sensors provide data as a series of sensor events. These embedded sensors are widely used in many functions in third-party applications. However, third-party applications can read data from embedded sensors without claiming any permissions. This may lead to security issues. Users’ privacy can be compromised by well-designed malicious applications that exploit embedded sensors. A compromised device due to a successful ransomware attack could potentially have broader security implications. If the ransomware is part of a more sophisticated attack, it might seek additional permissions or exploit vulnerabilities in the device’s operating system. This could indirectly impact various functionalities, including sensors. An attacker might attempt to manipulate the sensors for unauthorized access or surveillance. By successfully detecting ransomware attacks, the attack surface can be reduced. Additionally, this will limit unauthorized access to Android sensors.

There are different types of analyses of features for ransomware. Static analysis depends on features gathered without running any code, whereas dynamic analysis derives features based on code execution (or emulation). Static analysis of an Android application can depend on features extracted from either the manifest file or the Java bytecode. Since no code execution is necessary, static analysis is often seen to be more efficient. Meanwhile, features like dynamic code loading and system calls that are gathered while the application is running can be dealt with through dynamic analysis of an Android application. Dynamic analysis is more informative because the executed code is evaluated [9]. Network traffic analysis within the context of dynamic analysis focuses on monitoring and analyzing the network traffic generated by an Android device to identify any suspicious patterns and malicious behaviors associated with ransomware activity. Network connections are often utilized by ransomware to transfer encryption keys, communicate with command-and-control servers, and assist in data exfiltration or payment procedures. By monitoring the data exchanged between the device and external entities, a network traffic analysis seeks to detect these fraudulent activities. Lastly, hybrid analysis is a strategy for analysis that combines static and dynamic analysis to compensate for their individual shortcomings [10].

Since the preceding decade, substantial use of AI techniques, namely ML and DL, has been made in the cybersecurity discipline [11,12,13,14,15,16]. The potential of these techniques is to learn from the data that are provided and, as a result, extract valuable insights and correctly predict cases in the future. By enabling automated and intelligent analysis of intricate patterns, features, and behaviors within data, ML and DL play a significant role in malware detection [17,18,19,20,21,22,23]. These approaches enable security systems to accurately detect and classify malware with high accuracy, even as malware evolves and becomes more sophisticated. An effective solution to protect users against specifically ransomware attacks is ML-based techniques [24]. ML can be used to detect anomalous activities that are associated with ransomware attacks. ML-based security solutions can be utilized to analyze events across the network and alert administrators of the system about possible attack threats. Moreover, ML algorithms can learn and analyze past ransomware attacks data and build models that are capable of better predicting similar future attacks, which eventually helps to increase the security of systems. While ransomware has been a persistent global threat, it has continued to evolve, making traditional detection methods like signature-based approaches insufficient. As a result, researchers and security experts have turned to ML as a promising alternative for detecting and mitigating ransomware attacks. ML techniques have gained attraction in the realm of Android ransomware detection and classification [25]. These approaches leverage features, such as network traffic patterns, system calls, and file entropy to distinguish normal behaviors from malicious activities [26]. DL methods, in particular, have demonstrated remarkable efficacy in identifying various forms of malware, including ransomware, in addressing the dynamic nature of these threats.

While previous research has explored ML-based techniques for Android ransomware detection, there is still a need to comprehensively investigate their effectiveness and accuracy. As ransomware attacks on Android devices continue to rise, an ML-based model holds the potential to detect and prevent such attacks effectively. How effective is an ML-based model at detecting Android ransomware? This research question is crucial, as ransomware attacks on Android devices are becoming increasingly common, and an effective ML-based model can potentially help in detecting and preventing such attacks. Therefore, this study aims to assess the efficacy of ML and DL methods in identifying Android ransomware.

This study specifically concentrates on DT, SVM, KNN, an ensemble of these three algorithms (DT, SVM, KNN), FNN, and TabNet, utilizing a recent dataset readily available on Kaggle. KNN is a straightforward instance-based ML algorithm that is applied for classification and regression purposes. It categorizes data points based on the majority class of their K-nearest neighbors in the feature space [27]. In contrast, DT is a supervised learning algorithm that constructs a tree-like structure to make decisions [28]. SVM, on the other hand, is a robust classification and regression algorithm that identifies the hyperplane that optimally separates classes in high-dimensional space and can accommodate non-linear data through kernel functions [29]. FNN, also recognized as a multilayer perceptron (MLP), represents an artificial neural network (ANN) with unidirectional information flow, spanning multiple layers of interconnected neurons, and it serves various ML tasks [30]. Finally, TabNet is a specialized deep neural network model developed for tabular data, encompassing structured information commonly found in spreadsheets. It performs exceptionally well in capturing intricate relationships within the data by combining decision trees and attention mechanisms [31]. In aligning with our comprehensive approach, we utilized an ensemble model, combining DT, SVM, and KNN models. This strategic integration allows us to harness their collective decision-making capabilities, enhancing the model’s ability to capture diverse aspects of the data and yielding robust results.

The key contributions of this paper are as follows:

- Utilizing a real-world dataset comprising previously unused samples of well-known ransomware variants. This authentic dataset enhances the reliability of the evaluation, as it represents the actual scenarios encountered by users, making the results more applicable and meaningful.
- Identifying the most significant traffic features that contribute to Android ransomware detection.
- Introducing an ensemble model that combines the strengths of multiple ML algorithms. This ensemble approach aims to improve overall detection accuracy and robustness against evolving ransomware threats by leveraging the diverse perspectives and decision-making strategies of each individual model.
- Exploring and assessing the efficacy of two DL models which have not been deployed in the existing literature in the domain of Android ransomware detection.

The remaining part of this study is organized as follows: Section 2 provides a review of related literature in this domain. Section 3 presents the proposed methodology, including dataset description and the pre-processing phase, classification phase, and evaluation phase. Section 4 describes the experimental setup and optimization strategies. Section 5 presents the results and discussion. Section 6 highlights the limitations and recommendations. Section 7 presents the future work, while Section 8 contains the conclusions derived from this research study.

## 2. Review of Related Literature

This section presents the studies in the existing literature for the time period of 2018–2022 that utilized ML and DL techniques in the domain of Android ransomware detection.

Most of the studies deployed various ML techniques. Firstly, Khammas [32] proposed a byte-level static analysis for detecting ransomware attacks using the random forest (RF) model. A dataset consisting of 1680 executable files (840: ransomware, 840: benign) was used to train and evaluate their approach. The dataset was split into 50:50 training–testing sets with the same number of ransomware and goodware files to avoid imbalances. The proposed system used the gain ratio (GR) feature selection method to find the optimal dimension size between 1000 and 7000 features to build the RF model. It was found that constructing the RF model with 1000 features and 100 trees provided the best results in terms of time complexity and accuracy, with a prediction time of 1.37 s and an accuracy of 97.74%. A high number of trees can be considered a limitation to this study given the increased complexity related to it.

Masum et al. [33] proposed an ML-based approach for detecting and classifying ransomware attacks using a dataset with 54 features and 138,047 samples (96,632: ransomware, 41,415: benign). Of the samples, 70% of the used dataset were ransomware and the remaining 30% were legitimate, showing a clear imbalance issue. However, the authors focused on feature selection methods, including the variance threshold and variance inflation to eliminate low-variant and highly correlated features. Eventually, 12 features were selected to examine five different algorithms, namely decision tree (DT), RF, naive Bayes (NB), logistic regression (LR), and ANN for classification. The results demonstrated that the RF classifier outperformed the other models by achieving the highest accuracy of 99%, recall of 97%, F-beta of 97%, and precision of 99%.

Victoriano [34] and Ferrante et al. [35] employed the HelDroid dataset to distinguish Android ransomware from other malware. In Victoriano’s work, the dataset initially contained 1923 Android applications, and feature extraction resulted in 11 features. Utilizing DT, RF, gradient boosting (GB), and Adaboost classifiers, the DT classifier stood out with the highest accuracy of 98.8% and a low false positive rate (FPR) of 0.2%. Building on this dataset, Ferrante et al. proposed a hybrid system for ransomware detection, classifying 50 features with the J48, NB, and LR algorithms. The hybrid system achieved a precision of 100% and an FPR less than 4%, emphasizing the importance of combining static and dynamic analyses. Both studies highlight the need for more extensive and diverse datasets to validate the effectiveness of their approaches in covering all ransomware and legitimate applications.

Alsoghyer et al. [36] aimed to provide a comprehensive solution to tackle the increasing threat of Android ransomware. The dataset collected from different sources consisted of 1000 applications (500: ransomware, 500: benign). The authors used 116 permissions as a feature obtained through static analysis. Four ML models were used for classification, namely RF, J48, NB, and sequential minimal optimization (SMO). RF outperformed, with an accuracy of 96.9%. The proposed model had the potential to be a valuable addition to the existing ransomware detection systems. However, a limitation to be addressed is the effectiveness of this model, which may be reduced if ransomware creators find ways to bypass the requested permissions or if new types of ransomware emerge that require different detection methods. Further research is needed to address these potential limitations and improve the overall security of Android devices.

Consequently, Alzahrani et al. [37] presented a new computerized, lightweight approach known as RanDroid for identifying and mitigating ransomware threats on Android devices based on static and dynamic analyses. RanDroid extracted images and text to determine the existing locking screens or threatening notes. The authors used a dataset of 1450 applications (950: ransomware, 500: benign). NB was used for classification, which achieved an accuracy of 91%. This study provided promising results for using NB in detecting ransomware, but further work is needed to enhance its accuracy and expand its capabilities. Additionally, incorporating advanced techniques such as natural language processing (NLP) and spelling auto-correction can improve the model’s performance.

Additionally, Abdullah et al. [38] presented an Android ransomware detection approach utilizing dynamic analysis. The authors combined a public dataset, namely VirusTotal, and benign applications from Google Play. Eventually, it consisted of 800 applications (400: ransomware, 400: benign). A total of 52 system calls were obtained as features through dynamic analysis. Three ML models were used for classification, namely RF, J48, and NB. RF outperformed, with a true positive rate (TPR) of 98%, FPR of 1.6%, and accuracy of 98.31%.

Almomani et al. [39] proposed a ransomware detection approach for Android Version 11, API Level 30. The authors used crawlers for benign applications from Google Play, while ransomware applications were collected from the public dataset RansImDS-API & Permissions. A total of 302 permissions and API package calls were considered as features and used to parse 1000 applications (500: ransomware, 500: benign). Furthermore, GR was applied to select the top 225 features. The ML classifiers utilized were RF, DT, SMO, and NB. Comparatively, RF obtained the highest performance, with an accuracy of 98.2%, recall of 99%, and precision of 97.4%. It was found that in terms of size, DT was the least complex model.

However, Gera et al. [40] suggested a hybrid approach for detecting and eliminating Android ransomware. To extract the dominant feature set, this study utilized a brand-new dominant feature selection algorithm, which eventually selected the 60 most dominant and predictive features in the set. They collected 3249 application samples (1763: ransomware, 1486: benign). They used multiple classifiers during their experiment, such as J48, logistic model tree (LMT), RF, and random tree (RT). The study’s findings demonstrated that their hybrid approach had a 99.85% accuracy rate for detecting Android ransomware. Utilizing the suggested dominant feature selection method to choose the most relevant features was found to significantly improve classification. However, there were several limitations in this study as well, including the small sample size and lack of variety in the ransomware families examined.

Interestingly, a scalable framework known as an application programming interface-based ransomware detection system (API-RDS) was developed by Alsoghyer et al. [4] to detect ransomware applications. A dataset of 1000 applications (500: ransomware, 500: benign) was utilized for HelDroid and RansomProper projects, Virus Total, Koodous, and Google Play. With the help of the Weka platform, the API-RDS predictive model was built. The feature vector consisted of 174 features of API package calls belonging to Android API 27. Consequently, the data were partitioned using the 10-fold cross-validation technique. The classifiers RF, SMO, J48, and NB were deployed in the first experiment. RF outperformed, with an accuracy of 97%, area under the curve (AUC) of 99.5%, and kappa of 94%. Furthermore, in the second experiment, the framework successfully detected 96% of unseen ransomware samples and 97% of unseen benign samples, with an overall accuracy of 96.5%. It was discovered that java.lang with an average of 15,928.96 calls was the most commonly requested and used package in ransomware applications.

Additionally, a model was presented by Bagui et al. [41] for an Android ransomware intrusion detection system. A dataset consisting of 10,854 samples (4354: ransomware, 6500: benign) was utilized from CICAndMal2017. Ten ransomware families consisting of 80 features were considered and fed into the models. Three classifiers, J48, NB, and OneR, were deployed. J48 showed the best results, with a precision of 75.76%, recall of 75.73%, and F-score of 75.7%. Moreover, using the J48 classifier provided a decreased execution time with a lower number of attributes. The top 10 attributes in each of the ten ransomware families were found to include Init Win bytes backward and Init Win bytes forward.

Sharma et al. [42,43,44] collaborated using the RansomProber dataset for their Android ransomware detection studies. In their initial work, the RansomDroid framework employed unsupervised ML on 2300 Android package kits (APKs), achieving a 98.08% accuracy using the Gaussian mixture model (GMM). Extending their research to detect locker and crypto ransomware, they achieved a 99.59% accuracy on a dataset of 4076 applications utilizing the LR model. In a subsequent study, the authors developed a framework for classifying benign and malicious Android apps. Using a dataset of 4721 applications, the ensemble RF model achieved an accuracy rate of 99.67% in the binary classification task, demonstrating its potential for real-time identification of ransomware apps on Android-based handsets. The feature set comprised 1045 features across these studies.

Almomani et al. [45] proposed a novel approach to ransomware identification that relies on an ML technique that is based on evolution. The suggested method’s synthetic minority oversampling technique (SMOTE-tBPSO-SVM) employed the BPSO as the optimization and search algorithm and the linear SVM for classification and detection. They collected 10,153 application samples (500: ransomware, 9653: benign) from different data sources. Using 182 permissions and API calls as features, the performance of the suggested approach SMOTE-tBPSO-SVM outperformed conventional ML algorithms in terms of sensitivity (96.4%), specificity (99.9%), and g-mean (97.5%).

To address the limitations of the low detection accuracy and high computational complexity in existing solutions for Android ransomware detection, Hossain et al. [46] proposed a new method based on traffic analysis. The proposed method used the CICAndMal2017 dataset, which contains 84 features for four types of malwares, including ten types of ransomware attacks. Pre-processing techniques were applied to normalize and oversample the benign traffic, which resulted in a dataset of 402,834 instances. Afterward, a core step in this study employed particle swarm optimization (PSO) to select traffic features for binary (26) and multi-class (23) classification. Multiple experiments were applied, analyzed, and compared using DT and RF classifiers and different subsets of the selected features to perform detection on two levels: binary classification as ransomware or benign or multi-class classification of the ten types of ransomware traffic. RF showed the best performance in detecting ransomware, with an accuracy of 81.58%, whereas DT was found to be the best for multi-class classification.

Lastly, only one study employed DL, using the same dataset as [46]. Bibi et al. [47] proposed a DL-based method for ransomware detection in an Android environment by using an LSTM model. In the pre-processing phase, eight feature selection techniques were applied using the WEKA tool, and the most predictive 19 features were selected. A balanced 40,000 samples (20,000: benign, 20,000: ransomware) were considered for experimentation. They split the data into 80:20 training–testing sets. Then, numerous evaluation metrics were used to assess the performance of the model. The proposed model achieved an accuracy of 97.08% and is claimed to be capable, scalable, and proficient to detect ransomware attacks when placed at the Kernel of the Android OS.

Table 1 provided below presents a summary and comparison of the reviewed studies in the existing literature concerning the detection of Android ransomware, focusing on a wide range of ML [32,33,34,35,36,37,38,39,40,41,42,43,44,45,46] and DL [47] techniques, datasets utilized, number of samples, type of classification task (binary, multi-class), best-performing technique, and ultimately, the highest accuracy achieved in these studies.

Based on the reviewed literature, we found that a few limitations were more common and frequent than others. For instance, 71% of the studies had a small sample size issue [4,32,34,35,36,37,38,39,40,42,43,44]. All these studies had a sample size below 5 K, which, in consequence, has led these studies to apply ML techniques instead of DL, as the number of samples is not sufficient to build a DL model. We can remark that out of all the studies, only one study employed DL [47]. Only one study utilized unsupervised ML [42] as well. The reliance on anti-virus vendors to provide explicit labels to researchers can be an issue, especially when considering applying real-time solutions to detect Android ransomware attacks. Therefore, dependence on historical examples can cause the models to perform poorly when introducing an unknown attack, which can be resolved using unsupervised learning. Despite the excellent performance provided by DL models and unsupervised techniques, they are not adequately explored in recent literature. Therefore, there is an open area for research in applying DL and unsupervised solutions to Android ransomware detection. Although ML can be a viable solution in many cases, the dataset size is a critical factor. In this context, exploring the potential of DL becomes particularly relevant for its ability to handle complex patterns and relationships within the data, which may lead to more robust Android ransomware detection solutions that are especially noteworthy, as DL models are known for their scalability. This is why they can handle large datasets. Moreover, the curse of dimensionality was a common limitation for most of the studies [32,35,36,38,39,40,41,42,43,44,45]. These studies included 50 or more features in their experiments, which can be computationally expensive. Highly dimensional data can lead to increased complexity of the models utilized, as well as making the interpretation of the results challenging. Additionally, models built with high-dimensional data have a higher risk of overfitting, thereby being less reliable. Some studies have applied feature selection, but the number of features remained high. Furthermore, many studies [33,34,35,37,40,41,42,43,44,45,46] have an imbalanced distribution of class labels, which is common in anomaly detection problems. The “anomaly” cases tend to be much less than regular/normal cases. However, this issue must be resolved by under-sampling the majority class to be even with the minority class. Despite losing valuable data, this solution increases the performance and reliability of the models, as imbalanced data can lead to overfitting or bias. Notably, we can see that almost all the studies worked on binary classification instead of multi-class classification, except [46], which did both. Binary classification is an advantage, since binary classifiers have higher convergence rates. Moreover, few studies have explored the potential of traffic analysis [41,46,47], regardless of its promising results.

In this study, we aim to address these limitations by utilizing a large dataset based on traffic analysis to solve data imbalance issues. We shall fill the gap in the literature by deploying an ensemble model and two DL models which have not been deployed in the existing literature, in addition to other ML models. A smaller set of features shall be used to overcome the curse of dimensionality and reduce computational complexity. This will provide a cutting-edge solution to Android ransomware detection.

## 3. Methodology

### 3.1. Materials and Methods

**Figure 1:**
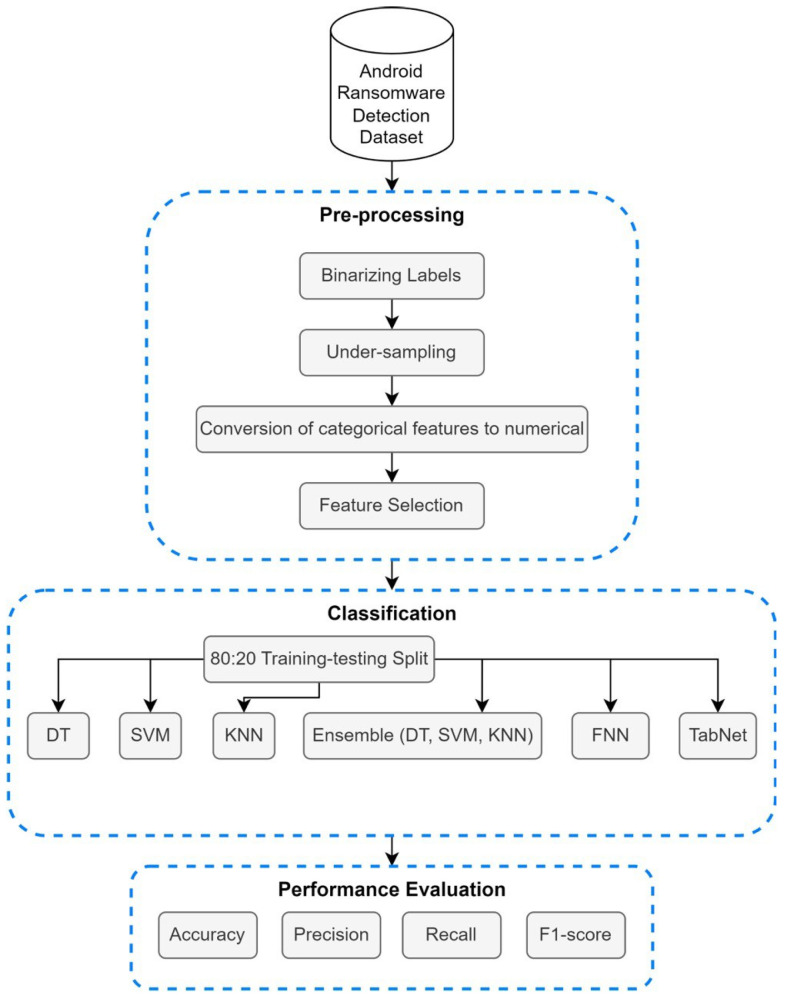
Proposed methodology pipeline.

In order to address the limitations in the existing literature and fill in the gap, this study examined and classified network traffic and classified it as ransomware or benign by utilizing a very recent dataset. Useful attributes were extracted from the dataset to achieve the best performance possible. This study was conducted using a consistent methodology, which included the following major steps: data acquisition, pre-processing, classification, and evaluation. When we acquired the dataset, we first visualized and assessed the data to understand the features present. Afterward, the pre-processing steps to improve the quality of the data and enhance the performance and reliability of the models were clear. The pre-processing steps included binarizing the labels, under-sampling, converting categorical features to numerical, and feature selection using forward feature selection and feature importance techniques. Then, the dataset was split into 80:20 training–testing sets, respectively, to prepare it for classification using DT, SVM, KNN, ensemble of (DT, SVM, KNN), FNN, and TabNet models. Lastly, the results were obtained and analyzed. Figure 1 presents a visual representation of the methodological steps adopted for this study.

### 3.2. Dataset Description

**Figure 2:**
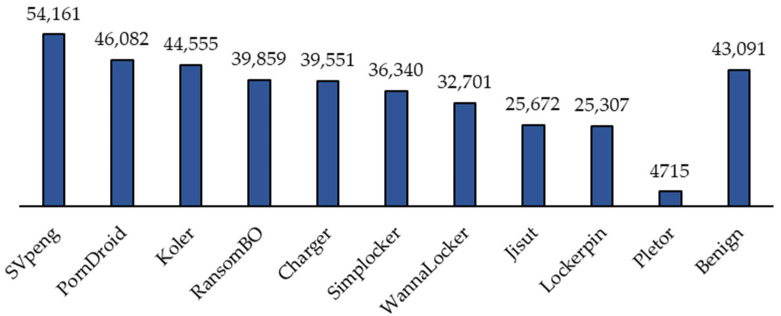
Distribution of class labels.

The dataset used in this study is a public dataset available on Kaggle, called Android Ransomware Detection, which has been collected and published by Subhadeep Chakraborty from the Canadian Institution for Cybersecurity (CIC) to detect ransomware attacks in Android networks using ML techniques (dataset link added below under “dataset availability”). The dataset is recent, and as far as we know, it has not been used before. Therefore, there is no benchmark work on the dataset to consider. The dataset contains 392,035 records and 85 attributes (including the class attribute). Additionally, the dataset does not contain any missing values. The dataset contains 10 types of Android ransomware and benign traffic (11 labels in total), as shown in Figure 2. Figure 2 demonstrates the high imbalance in the class label distribution in the dataset as well. Out of the 392,035 records, only 43,091 are benign traffic. The imbalance issue is extremely common when dealing with anomaly detection problems. Moreover, Table 2 shows all the features present in the dataset.

### 3.3. Pre-Processing Phase

In this phase, firstly, data visualization was done. We first checked for missing values, the distribution of classes, type of attributes (categorical/numerical), and correlation coefficients. It was observed that 12 attributes had their values as 0 for all samples in the dataset. Hence, they were deleted. This is because an attribute with exactly the same value for all samples (either zero or non-zero) does not contribute to the learning process of a model and might even confuse it. The following were the features that were deleted: Bwd PSH Flags, Fwd URG Flags, Bwd URG Flags, RST Flag Count, CWE Flag Count, ECE Flag Count, Fwd Avg Bytes/Bulk, Fwd Avg Packets/Bulk, Fwd Avg Bulk Rate, Bwd Avg Bytes/Bulk, Bwd Avg Packets/Bulk, Bwd Avg Bulk Rate. Furthermore, the feature “Time Stamp” was difficult to convert to a numerical or date–time object, which made it challenging to process. Thus, it was deleted. In total, 13 features were deleted for the reasons mentioned above. Furthermore, binarizing labels, under-sampling, and the conversion of categorical attributes to numerical attributes was performed and shall be elaborately explained in the sub-sections below.

#### 3.3.1. Binarize Labels

Originally, the dataset comprised 11 class labels including 10 types of ransomware attacks and the benign class. However, in this study, we aim to classify network samples as ransomware or benign regardless of the type of ransomware. Therefore, we binarized the multi-class labels to (ransomware, benign) only. The ransomware class was represented as 1 whereas the benign class was represented as 0.

#### 3.3.2. Under-Sampling

**Figure 3:**
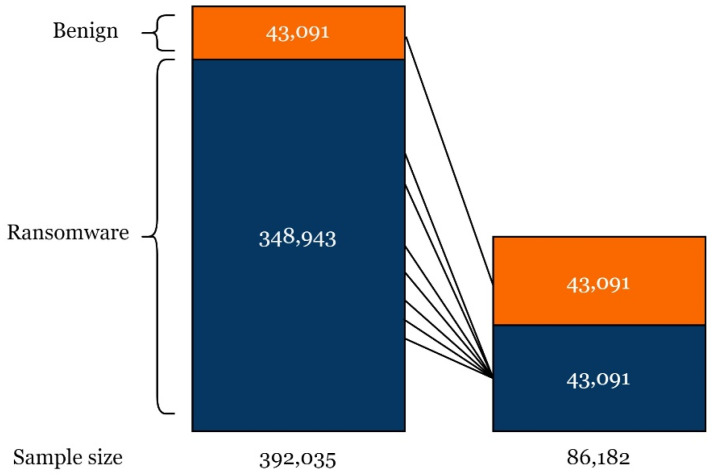
Under-sampling technique applied to the dataset.

After binarizing the labels, in order to address the class imbalance in the dataset, we employed a randomized under-sampling technique. This method is commonly utilized to equalize the class labels in a dataset by reducing the number of the significantly more prevalent class (ransomware) in our case to be equal to the minority class (benign). Therefore, both class labels were under-sampled to be of equal size (43,091: ransomware; 43,091: benign), as shown in Figure 3.

#### 3.3.3. Conversion of Categorical Attributes to Numerical Attributes

Numerical representations of categorical attributes provide a consistent and standardized way to represent the data. This enables more efficient comparisons, calculations, and transformations, which are essential for many ML techniques. Therefore, three categorical features, namely source IP, destination IP, and flow ID, were converted to numerical.

#### 3.3.4. Feature Selection

Feature selection is an important step in ML that involves identifying and selecting a subset of relevant features from the available set of input variables. It enables improved model performance, interpretability, efficiency, and generalizability. In this study, feature selection was performed using two techniques, which are forward feature selection and feature importance (k = 10). Forward feature selection is a feature selection technique that starts with an empty set of features and iteratively adds one feature at a time to build the best-performing subset. The process begins by evaluating the performance of the model using each individual feature and selecting the one that yields the highest performance. In each subsequent iteration, the algorithm adds one additional feature that provides the maximum improvement in model performance until a stopping criterion is met. A total of 22 features were obtained from the forward feature selection technique. On the other hand, the feature importance technique refers to the process of determining the relevance or contribution of each feature in an ML model. It helps identify the most important features that have the most significant impact on the model’s predictions. The feature importance score is typically calculated based on how much each feature reduces the impurity or error in the model when it is used for splitting decisions. The higher the feature importance score, the more influential the feature is in making accurate predictions. Using the feature importance technique, 32 features were obtained. After choosing the common features through manual selection, a total of 19 features for which the model performance was the best were selected. The final set of selected features is shown below in Table 3.

### 3.4. Classification Phase

ML is the subfield of AI where machines can learn and understand data without the need to be explicitly programmed. ML allows autonomous solutions for a wide range of problems faced in computation. Advancements in ML algorithms have led to the development of DL algorithms, which are more sophisticated and mathematically complex. ML can be supervised, semi-supervised, or unsupervised. In this study, we focused on supervised ML, as it is the best-suited for the obtained dataset. Three supervised ML models, along with an ensemble model and two DL models, were trained and tested. The models are DT, SVM, KNN, an ensemble model of (DT, SVM, KNN), FNN, and TabNet.

#### 3.4.1. Decision Trees

DTs are a supervised learning technique characterized by their non-parametric approach, which involves constructing a hierarchical structure of if–else conditions according to the properties of the input data. It acts as a predictor, denoted as h: X→Y, and it operates by traversing from the root node of the tree to a leaf node to predict the label associated with an input instance X. At each step along this path, the next child node is determined based on how the input space is partitioned. This partitioning process typically relies on the characteristics of X or a predefined set of rules for splitting [48,49].

#### 3.4.2. Support Vector Machines

SVMs are an effective supervised learning technique that may be utilized for both regression and classification applications. They works especially well for resolving difficult issues where there is a distinct line separating the classes. SVM operates by locating an ideal hyperplane that maximally separates the classes in the feature space. During training, SVMs determine the significance of each training data point in illustrating the decision border between two classes. Typically, only a part of the training points—those that are situated on the decision boundary and are known as support vectors—matter in identifying the decision border. To calculate a new position, the distance from each support vector is computed. A classification choice is made [50] based on the support vectors’ importance and the distances from them that were discovered during training.

#### 3.4.3. K-Nearest Neighbors

The KNN algorithm is considered one of the simplest ML algorithms. It is commonly used for classification problems but can be extended for regression problems as well. It is a non-parametric algorithm in which the majority class label determines the class label of a new data point among its nearest ‘k’ (where k is an integer) neighbors in the feature space. During training, KNN stores the labeled instances of the training data, which serve as the “knowledge” for making predictions. It receives input, calculates the distance, and finds neighbors. Lastly, as the k nearest neighbors are identified, the KNN algorithm assigns the class label to the new instance based on a majority voting scheme. The class label with the highest count among the neighbors is assigned as the predicted class for the new instance. In the case of a tie, the class label may be selected randomly or based on additional rules [50].

#### 3.4.4. Voting Classifier

Ensemble models are known to combine the predictions of multiple individual models to make more robust and accurate predictions. It is typically used for classification tasks where each individual classifier makes predictions on the input data, and the final prediction is determined by aggregating the votes of all the classifiers. The voting classifier can be used with different types of base classifiers, such as DT, SVM, KNN, or any other classifier that supports multi-class classification. It can also handle a mix of classifiers with different algorithms or parameter settings [51,52].

#### 3.4.5. Feedforward Neural Networks

FNNs are a type of ANN inspired by the human brain’s computational model. FNNs are structured as interconnected layers of neurons. FNNs consist of input, hidden, and output layers. The neurons’ interactions involve weighted connections and activation functions, enabling the network to model complex relationships. This structure underpins FNNs’ ability to approximate various functions, granting them universal function approximation capabilities [53].

#### 3.4.6. Tabular Attention Networks

TabNet is a specialized deep neural network for tabular data which adeptly handles classification and regression tasks [31]. Its architecture mirrors DTs, with a series of sub-networks for hierarchical decision-making. In each decision step, TabNet selectively processes feature subsets, dynamically determining attention through a learned mechanism borrowed from the transformer architecture [31]. Combined with the power of deep learning, TabNet is particularly effective for extracting patterns and making accurate predictions in tabular datasets.

### 3.5. Evaluation Phase

ML and DL classifiers can be compared using different performance metrics. This study employs accuracy, precision, recall, and F1-score to evaluate and contrast the effectiveness of the deployed models. The confusion matrix, comprising predicted and actual classifications in a four-way table, is utilized to assess the accuracy of the classifier.

(i) True positive (TP): correctly predicting Ransomware Android network traffic as Ransomware.
(ii) False positive (FP): incorrectly predicting Ransomware Android network traffic as Benign.
(iii) True negative (TN): correctly predicting Benign Android network traffic as Benign.
(iv) False negative (FN): incorrectly predicting Benign Android network traffic as Ransomware.

## 4. Experimental Setup

The experiments were carried out using Python version 3.5 on Kaggle with a GPU P100. The device used was a Windows 11 Home operating system with 8 GB RAM and core i7. The Android Ransomware Detection dataset publicly available on Kaggle was used after under-sampling. The dataset now consisted of 43,091 ransomware and 43,091 benign samples. After applying feature selection techniques (forward feature selection and feature importance with k = 10), the best 19 common features were selected to train and test the models. For training and testing, an 80:20 split of the data was performed. Two experiments were conducted in total, using DT, SVM, KNN, an ensemble model of (DT, SVM, KNN) with random_state = 42, FNN, and TabNet. In experiment 1, all 70 features were utilized. In experiment 2, the best 19 features were utilized. The DT model was optimized using hyperparameter tuning, and then Grid Search with Cross Validation was applied. For the ensemble model, firstly, the votingClassifier was imported from sklearn.ensemble. Then, the ensemble model was built and passed to the votingClassifier. We encountered the challenge of a high training loss during the model training process, indicating that the model was having difficulty effectively learning from the training data and reducing the disparity between its predicted outputs and the actual target values. This issue was evident, as the loss value consistently increased with each epoch. To address this problem, we applied feature scaling techniques to the input data. Feature scaling aims to normalize the range of input features, ensuring that they are on a similar scale. The model container was initialized as sequential. The detailed parameter settings applied to all models are shown in Table 4 below.

## 5. Results and Further Discussion

In the two experiments conducted, four evaluation metrics—accuracy, precision, recall, and F1-score—were recorded, and the models were evaluated using them.

Table 5 shows the results obtained for the DT, SVM, KNN, ensemble model of (DT, SVM, KNN) with random_state = 42, FNN, and TabNet models for experiment 1 and experiment 2. In experiment 1, all 70 features were utilized. In experiment 2, the best 19 features were utilized. In terms of accuracy, the highest accuracy of 97.24% was achieved by DT, followed by Ensemble, FNN, SVM, TabNet, and lowest by KNN of 88.43%. In terms of precision, DT achieved the highest value, i.e., 98.50%, and lowest of 88.96% by TabNet. Recall is a metric that accesses the model’s sensitivity by quantifying the model’s ability to correctly identify positive instances from the total number of actual positive instances in the dataset. Therefore, recall is an important evaluation metric for our specific problem. All the models showed a recall greater than 97%. The SVM achieved an outstanding recall of 100%, and TabNet showed the least, i.e., 97.28%. Taking the F1-score into consideration, the DT model achieved the highest score of 98.45%, and the lowest was 93.77% by KNN.

**Figure 4:**
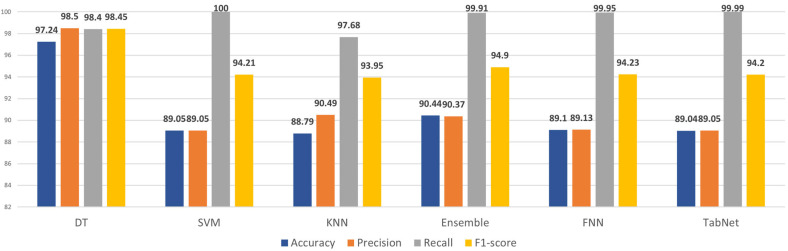
Results comparison.

The DT and FNN model showed better performance in experiment 2, i.e., after feature selection. In the case of SVM, it can be seen that the results obtained before and after feature selection remained the same. SVM automatically takes feature importance into account while building a model. It looks for the optimum hyperplane that maximizes the separation between the data points from various classes. Higher weights are assigned to features that contribute more to class separation. This characteristic of SVM can reduce the need for explicit feature selection. Moreover, another possibility could be due to the curse of dimensionality. SVM usually handles large datasets with numerous features well. It effectively performs in high-dimensional spaces. It is able to deal with inherent noise and redundancy in high-dimensional feature spaces; therefore, feature selection may not be highly critical in such cases. KNN experienced a slight decrease in performance in experiment 2 after applying feature selection techniques. Regarding the ensemble model, despite it being able to learn and automatically extract the most relevant features without the need for explicit feature selection, the performance marginally decreased. TabNet’s performance declined to some extent in experiment 2 after feature selection. An infographic shown in Figure 4 has been provided for a better visualization and comparison of the results of all the models.

**Figure 5:**
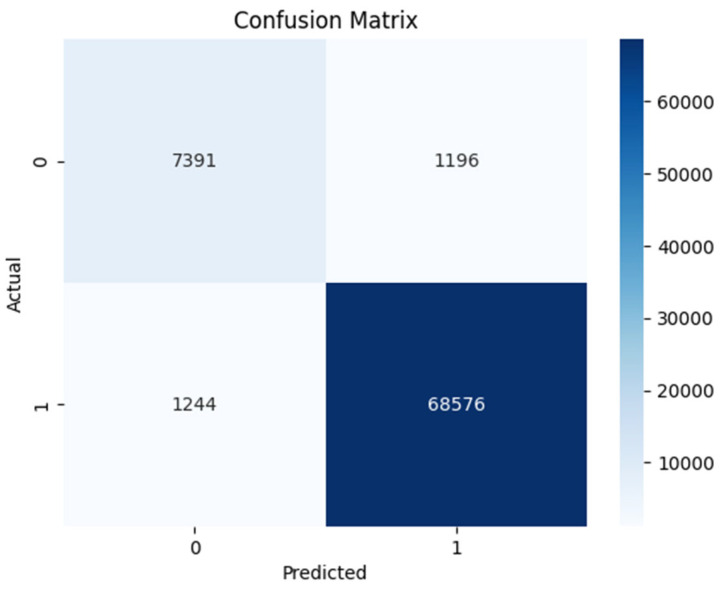
Experiment 1—DT confusion matrix.

**Figure 6:**
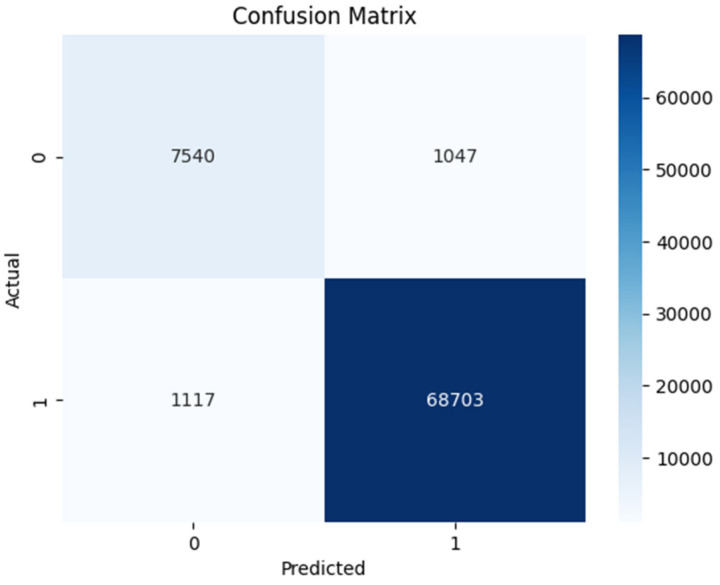
Experiment 2—DT confusion matrix.

The confusion matrices for DT shown in Figure 5 and Figure 6 were analyzed. The correct identification of benign Android network traffic is considered a TN; however, correct identification of Android ransomware traffic is a TP. Incorrect identification of benign network traffic as ransomware is a FN, and incorrect identification of ransomware as benign network traffic is a FP. If a model has more TPs and TNs (or fewer FNs and FPs), it is considered more accurate. In the figures for the results of the DT classifier, we can see that the numbers of instances of TPs and TNs are more. It achieved the highest accuracy, precision, and F1-score. The DT model is effective for binary classification and was able to handle a large numerical values dataset. Before the optimization, the DT model achieved an accuracy of 97.23%, precision of 98.50%, recall of 98.39%, and an F1-score of 98.44%. However, through the application of hyperparameter tuning using grid search, minor improvements were observed in the model’s performance. The optimized DT model (with 19 features) exhibited an accuracy of 97.24%, precision of 98.50%, recall of 98.40%, and an F1-score of 98.45%. These results clearly demonstrate the effectiveness of hyperparameter tuning in enhancing the overall performance of DT.

Overall, the DT performed the best in terms of accuracy, precision, and F1-score while requiring the shortest execution time of 3–4 min. The DT is good at capturing non-linear relationships and handling complex feature interactions. Among the ML models, SVM required the longest execution time of 1 h and 30 min but gave the best recall of 100%. This can be considered a major drawback in real-time attack detection. However, SVM is powerful in finding optimal hyperplanes for separating classes in high-dimensional spaces. KNN is effective in identifying local patterns and can handle diverse data distributions. In the existing literature, only two studies used ensemble models. Sharma et al. [44] used an RF ensemble model, and Almomani et al. [45] combined BPSO, SVM, and SMOTE. Ferrante et al. [35] and Gera et al. [40] made use of a hybrid model. J48, NB, and LR were combined by Ferrante et al. [35]. RF, J48, LR, and RT were combined by Gera et al. [40]. This shows that ensemble models have not been explored enough in the domain of Android ransomware detection, emphasizing the need for further investigation into the potential benefits for enhanced model performance. Examining ensemble learning in this context proves to be significant, as it allows us to leverage the strengths of multiple base models, enhancing the overall effectiveness of the ransomware detection system. This was the primary driving force to carry out an experiment to highlight the diversity among the base models (DT, SVM, KNN) and implement hard voting to build an ensemble model. Each model individually has its own strengths and weaknesses. Therefore, in order to fill the existing gap in literature, we utilized an ensemble model. By combining the DT, SVM, and KNN models using hard voting, it was seen that we are able to benefit from their collective decision-making and increase the chance of capturing different aspects of the data, thereby acquiring good results. The proposed ensemble model was able to perform well on our utilized large dataset. The studies in the literature that used ensemble models achieved an accuracy of >99%. However, they used small datasets, which had the data imbalance issue; therefore, there is a possibility that these models suffered from overfitting. Recall is an important evaluation metric with respect to the specific problem being addressed in this study. The proposed ensemble model achieved a very high recall of 99.93%, with only 19 best features.

The results achieved by the two DL models are assessed and compared. FNN was used, as it is more suitable for the data type present in the used dataset. Moreover, we deployed it for our tabular dataset because our dataset size was large enough, i.e., approximately 90 K. It achieved a recall of 99.95%, which demonstrates that DL models which have not been sufficiently explored in the literature hold the capacity to show remarkable performance. FNN is considered as an out-of-date and a simple three-layer model, whereas TabNet is seen as a state-of-the-art model. Nonetheless, FNN demonstrated a better accuracy, precision, and F1-score than TabNet. FNN showed robust performance with all the features and the best 19 features. TabNet performed well with all the features, but with the best 19 features, there was a noticeable drop in its performance. When comparing the nature of the models, the FNN model can be adapted using various data modalities including images, text, and sequences. On the other hand, TabNet is specifically designed for tabular datasets, making it well-suited for our dataset. In terms of execution time, FNN required less than 1 h, whereas TabNet required a longer period of 6.5 h. Furthermore, by parallelizing the code, it was only reduced to 4 h. Given the nature of the dataset utilized and the need to obtain timely results, FNN emerges as the preferable choice due to its quick execution, aligning well with the requirements for rapid and efficient performance in our chosen research subject. Despite FNN being considered outdated, its practicality in meeting the specific demands of our research subject outweighs TabNet. This emphasizes the importance of considering model relevance and applicability over recency in certain contexts.

In terms of quantitative evaluation, the FPR values for KNN, the ensemble model, FNN, and TabNet are high. These models are incorrectly predicting a certain number of instances as ransomware when those instances are benign. However, all the models have extremely low false negative rate (FNR) values, emphasizing their efficacy in detecting ransomware instances. A low FNR is crucial in the context of security, as missing a true ransomware threat could have severe consequences.

In the context of Android ransomware detection, this study primarily focused on the analysis of network traffic data. However, it is noteworthy that Android devices come equipped with diverse built-in sensors, such as accelerometers, gyroscopes, proximity sensors, microphones, and temperature sensors. By implementing a security solution for ransomware detection, this study extends to protect not only the device’s core functionalities but also the Android sensors. Although our current work did not involve the integration of sensor data, the potential role of sensors in enhancing ransomware detection deserves attention. Sensors can provide valuable insights into the physical context and user interactions with the device. In addition to enhancing the accuracy and adaptability of ransomware detection systems against emerging threats, the combination of network traffic analysis and sensor data holds promises for establishing a dynamic and resilient defense mechanism.

Lastly, it is essential to consider the comparative findings from previous research. For instance, in the study referenced as [34], DT achieved an accuracy rate of 98.8% with 1923 records. In contrast, our study, which employed a dataset encompassing 392,035 records, attained a commendable accuracy rate of 97.24%. Turning our attention to ensemble models, ref. [44] demonstrated remarkable success, achieving a high accuracy of 99.67% when utilizing a dataset consisting of 4721 records. Similarly, in ref. [45], an exceptional accuracy of 99.9% was recorded using a dataset comprising 10,153 records. Within the scope of our study, employing a substantial dataset of 392,035 records, we achieved a competitive accuracy level of 90.4%. These comparative findings accentuate the pivotal role played by dataset size in influencing the performance of ML models.

## 6. Limitations and Recommendations

This study was conducted in a simulated environment rather than a real-world scenario. It is essential to assess the model’s robustness in the real world against adversarial attacks. Moreover, there was a lack of explainability for the models’ obtained results. Techniques such as local interpretable model–agnostic explanations (LIME) or Shapley additive explanations (SHAP) can be applied to post hoc interpretability. Android devices have limited resources and computational capabilities, so the ML models designed for ransomware detection need to be lightweight and efficient to run on these devices without causing performance degradation. Lightweight machine learning could be a potential solution, as this involves the building of compressed ML models which are suitable for execution on Android-based edge devices. Moreover, extracting relevant features from Android applications can be complex. Choosing appropriate features and extracting them accurately is vital for the model’s performance. It requires domain expertise and knowledge of Android malware characteristics. Since feature selection techniques may be necessary to reduce dimensionality and remove irrelevant features, this could affect the model’s performance if not done correctly. From a temporal aspect, ransomware is continually evolving, with new variants and evasion techniques emerging regularly. A model trained on a specific set of ransomware samples may not be effective in detecting newly developed ransomware strains. Regular updates and continuous retraining of the model are necessary to keep up with the evolving threat landscape.

## 7. Future Work

With regards to future work, we shall attempt to assess the effectiveness of other feature selection methods in addition to using unsupervised algorithms. Additionally, we utilize a larger dataset by combining multiple datasets of more attacks and performing real-time attack detection. Our current study primarily focuses on network traffic data analysis for Android ransomware detection, with a focus on the potential benefits of integrating sensor data. Android devices have various built-in sensors, such as accelerometers, global positioning system (GPS) gyroscopes, proximity sensors, microphone and audio sensors, and temperature sensors. These sensors record data about the physical surroundings of a device and how it interacts with the environment. Sensors can play an important role in dynamic analyses, and combining sensors’ data with network traffic analyses can lead to a promising multimodal approach in Android ransomware detection. Sensor data can be gathered over time to build a baseline of typical device behavior. The statistical features of sensors, such as the standard deviation, variance, mean, and entropy of sensor readings, can be extracted to detect deviations from the baseline behavior. This approach leverages the device’s physical context and network traffic behavior and can significantly improve the accuracy and adaptability of ransomware detection systems against emerging ransomware threats. Future research should explore this integrated approach. Collaborations between sensor data analysis and cybersecurity experts could advance the state-of-the-art in Android ransomware detection and ensure more robust mobile device security.

## 8. Conclusions

In recent times, Android has been experiencing an upsurge in terms of devices, users, and technology, making our day-to-day activities simpler and faster. Nevertheless, ease often accompanies insecurity, which raises many privacy and security concerns. These concerns mainly include cyberattacks, which require very careful handling. In order to address the above-mentioned concerns, this research study attempted to fulfil the current need and aimed to detect Android ransomware attacks by using ML- and DL-based techniques. Firstly, an inclusive literature review was provided to showcase the existing studies in order to analyze gaps and find new research directions. A very recent Android ransomware detection dataset, 2023 from Kaggle, was utilized to carry out two experiments. As part of data pre-processing, a randomized under-sampling technique was adopted to resolve the dataset imbalance. After data pre-processing, feature selection was applied, using forward feature selection and feature importance. A total of 19 features were found to be crucial for analysis and attack identification. After feature selection, a dataset split of 80:20 was performed for training and testing. Two experiments were conducted by deploying DT, SVM, KNN, an ensemble model of (DT, SVM, KNN), FNN, and TabNet. In experiment 1, all 70 features were utilized. In experiment 2, the best 19 features were utilized. The performance of these models was calculated in terms of accuracy, precision, recall, and F1-score. As a result, DT outperformed, with an accuracy of 97.24%, precision of 98.50%, and F1-score of 98.45%. The highest recall of 100% was obtained using the SVM model. The confusion matrices for the DT were also analyzed. The findings of this study contribute to advancing the field of Android ransomware detection by providing valuable insights and securing Android devices in the face of evolving cyber threats.

## Figures and Tables

**Table 1:** Summary of the reviewed findings.

Ref.	Year	Dataset	Analysis Type	Features Set	No. of Features	No. of Samples	Balanced	Binary/ Multi Class	Best-Performing Technique	Accuracy
[32]	2020	Khammas-2020	Static	Executable files	1000	1680	Yes	Binary	RF	97.74%
[33]	2022	Mathur-2020	-	-	12	138,047	No	Binary	RF	99%
[34]	2019	HelDroid	Static, dynamic	Android applications	11	1923	No	Binary	DT	98.8%
[35]	2018	HelDroid	Static, dynamic	Android applications	50	3058	No	Binary	Hybrid model	Precision (100%)
[36]	2020	HelDroid RandomProber Varus Total Koodous	Static	Permissions	116	1000	Yes	Binary	RF	96.9%
[37]	2018	ID-ransomware.RU Contagio Malware Dump theZoo	Static, dynamic	Android applications	-	1450	No	Binary	NB	91%
[38]	2019	VirusTotal Google Play	Dynamic	System calls	52	800	Yes	Binary	RF	98.31%
[39]	2021	RansImDS-API& Permissions	Dynamic	Android Applications (permissions, API calls)	225	1000	Yes	Binary	RF	98.2%
[40]	2021	AMD	-	Android applications	60	3249	No	Binary	Hybrid model	99.85%
[4]	2019	HelDroid RansomProper Virus Total Koodous Google Play	Static, dynamic	Android applications (API calls)	174	1000	Yes	Binary	RF	97%
[41]	2021	CICAndMal-2017	Traffic	Network traffic	80	10,854	No	Binary	J48	Precision (75.76%)
[42]	2021	RansomProber Androzoo	Static	APKs	1045	4300	No	Binary	GMM	98.08%
[43]	2020	RansomProber AndroZoo	Static	APKs	1045	4076	No	Binary	LR	99.59%
[44]	2021	RansomProber AndroZoo	Static	APKs	1045	4721	No	Binary	Ensemble model	99.67%
[45]	2021	RansImDS	Dynamic	Android applications (permissions, API calls)	182	10,153	No	Binary	Ensemble model	99.9%
[46]	2022	CICAndMal-2017	Traffic	Network traffic	26–23	402,834	No	Binary, multi	RF	81.58%
[47]	2019	CICAndMal-2017	Traffic	Network traffic	19	40,000	Yes	Binary	LSTM	97.08%

**Table 2:** Features found in the dataset.

No.	Feature	No.	Feature	No.	Feature
1.	Flow ID	30.	Fwd IAT min	58.	Subflow bwd packets
2.	Source IP	31.	Bwd IAT total	59.	Subflow bwd bytes
3.	Source port	32.	Bwd IAT mean	60.	Init_Win_bytes_forward
4.	Destination IP	33.	Bwd IAT std	61.	Init_Win_bytes_backward
5.	Destination port	34.	Bwd IAT max	62.	act_data_pkt_fwd
6.	Protocol	35.	Bwd IAT min	63.	min_seg_size_forward
7.	Flow duration	36.	Fwd PSH flags	64.	Active mean
8.	Total fwd packets	37.	Fwd header length	65.	Active std
9.	Total backward packets	38.	Bwd header length	66.	Active max
10.	Total length of fwd packets	39.	Fwd packets/s	67.	Active min
11.	Total length of bwd packets	40.	Bwd packets/s	68.	Idle mean
12.	Fwd packet length max	41.	Min packet length	69.	Idle std
13.	Fwd packet length min	42.	Max packet length	70.	Idle max
14.	Fwd packet length mean	43.	Packet length mean	71.	Idle min
15.	Fwd packet length std	44.	Packet length std	72.	Bwd PSH flags
16.	Bwd packet length max	45.	Packet length variance	73.	Fwd URG flags
17.	Bwd packet length min	46.	FIN flag count	74.	Bwd URG flags
18.	Bwd packet length mean	47.	SYN flag count	75.	RST flag count
19.	Bwd packet length std	48.	PSH flag count	76.	CWE flag count
20.	Flow bytes/s	49.	ACK flag count	77.	ECE flag count
21.	Flow packets/s	50.	URG flag count	78.	Fwd avg bytes/bulk
22.	Flow IAT mean	51.	Down/up ratio	79.	Fwd avg packets/bulk
23.	Flow IAT std	52.	Average packet size	80.	Fwd avg bulk rate
24.	Flow IAT max	53.	Avg fwd segment size	81.	Bwd avg bytes/bulk
25.	Flow IAT min	54.	Avg bwd segment size	82.	Bwd avg packets/bulk
26.	Fwd IAT total	55.	Fwd header length	83.	Bwd avg bulk rate
27.	Fwd IAT mean	56	Subflow fwd packets	84.	Time stamp
28.	Fwd IAT std	57.	Subflow fwd bytes	85.	Label
29.	Fwd IAT max				

**Table 3:** Final set of features selected.

No.	Feature	No.	Feature	No.	Feature
1.	Flow ID	8.	Fwd packet length max	15.	Active mean
2.	Source IP	9.	Fwd packet length min	16.	Active std
3.	Source port	10.	Bwd packet length min	17.	Active max
4.	Destination IP	11.	Init_Win_bytes_forward	18.	Idle mean
5.	Destination Port	12.	Init_Win_bytes_backward	19.	Idle std
6.	Protocol	13.	act_data_pkt_fwd		
7.	Flow duration	14.	min_seg_size_forward		

**Table 4:** Parameter settings applied to all models.

Model	Parameters	Optimal Value Chosen
DT	‘max_depth’	[None, 5, 10, 15]
	‘min_samples_split’	[2, 5, 10]
	‘min_samples_leaf’	[1, 2, 4]
SVM	C Kernel gamma	[0.1, 1, 10] [‘linear’, ‘rbf’, ‘poly’] [‘scale’, ‘auto’]
	Activation function in hidden layers	ReLU with 32 units
	Number of neurons in output layer	1
	Activation function in output layer	Sigmoid
FNN	Batch size	16
	Number of epochs	10
	Number of layers	3
	Number of decision steps in the network	64
	Dimension of the attention embedding	32
	Number of steps in the attention and aggregation blocks	5
TabNet	Gamma	1.3
	Number of independently trained feature transformers	2
	Number of shared feature transformers	2
	Momentum for batch normalization	0.02
	Maximum absolute value	2.0

**Table 5:** Results obtained after experiment 1 using all 70 features and experiment 2 using best 19 features.

	DT		SVM		KNN		Ensemble (DT, SVM, KNN)		FNN		TabNet	
	All	Best 19	All	Best 19	All	Best 19	All	Best 19	All	Best 19	All	Best 19
**Accuracy**	96.89%	97.24%	89.05%	89.05%	88.79%	88.43%	90.44%	90.24%	89.09%	89.10%	89.04%	86.84%
**Precision**	98.29%	98.50%	89.05%	89.05%	90.49%	90.10%	90.37%	90.17%	89.12%	89.13%	89.05%	88.96%
**Recall**	98.22%	98.40%	100%	100%	97.68%	97.74%	99.91%	99.93%	99.95%	99.95%	99.99%	97.28%
**F1-score**	98.25%	98.45%	94.21%	94.21%	93.95%	93.77%	94.90%	94.80%	94.22%	94.23%	94.20%	92.94%

## Data Availability

The dataset utilized for this study can be found at: https://www.kaggle.com/datasets/subhajournal/android-ransomware-detection (accessed on 23 March 2023).
